# Histological and ultrastructural comparison of cauterization and thrombosis stroke models in immune-deficient mice

**DOI:** 10.1186/1476-9255-8-28

**Published:** 2011-10-18

**Authors:** Silvia Mora-Lee, Ma Salomé Sirerol-Piquer, María Gutiérrez-Pérez, Tania López, Mayte Casado-Nieto, Carlos Jauquicoam, Gloria Abizanda, Miriam Romaguera-Ros, Ulises Gomez-Pinedo, Felipe Prósper, José-Manuel García-Verdugo

**Affiliations:** 1 Hematology and Cell Therapy Area, Clinica Universidad de Navarra and Division of Cancer, Center for Applied Medical Research (CIMA), University of Navarra, Pamplona, Spain; 2 Department of Comparative Neurobiology. Cavanilles Institute. CIPF. CIBERNED, Valencia, Spain; 3 Instituto de Investigación Sanitaria San Carlos (IdSSC), Madrid, Spain

**Keywords:** cerebral ischemia, scar, thrombosis, cauterization, SVZ, gliosis, inflammation

## Abstract

**Background:**

Stroke models are essential tools in experimental stroke. Although several models of stroke have been developed in a variety of animals, with the development of transgenic mice there is the need to develop a reliable and reproducible stroke model in mice, which mimics as close as possible human stroke.

**Methods:**

BALB/Ca-RAG2^-/-^γc^-/- ^mice were subjected to cauterization or thrombosis stroke model and sacrificed at different time points (48hr, 1wk, 2wk and 4wk) after stroke. Mice received BrdU to estimate activation of cell proliferation in the SVZ. Brains were processed for immunohistochemical and EM.

**Results:**

In both stroke models, after inflammation the same glial scar formation process and damage evolution takes place. After stroke, necrotic tissue is progressively removed, and healthy tissue is preserved from injury through the glial scar formation. Cauterization stroke model produced unspecific damage, was less efficient and the infarct was less homogeneous compared to thrombosis infarct. Finally, thrombosis stroke model produces activation of SVZ proliferation.

**Conclusions:**

Our results provide an exhaustive analysis of the histopathological changes (inflammation, necrosis, tissue remodeling, scarring...) that occur after stroke in the ischemic boundary zone, which are of key importance for the final stroke outcome. This analysis would allow evaluating how different therapies would affect wound and regeneration. Moreover, this stroke model in RAG 2^-/- ^γC ^-/- ^allows cell transplant from different species, even human, to be analyzed.

## Background

To date, with the exception of tissue-type plasminogen activator, there is no effective therapy for management of acute stroke. Several animal stroke models have been employed in order to develop new agents for treatment of ischemic stroke. However, despite providing important insights into the pathophysiology of the disease and numerous potential therapeutic targets, the translation of these results from bench to bedside has been disappointing [1].

A reliable in vivo animal model of stroke must reproduce the etiology, anatomical, functional and metabolic consequences of human disease as closely as possible. Different focal and global ischemic models have been developed and characterized in primates, dogs, gerbils [2-4] and rodents [1,5]. Consensus has emerged in favor of focal ischemic models and one of the most common approaches relays in the occlusion of the middle cerebral artery (MCAo). Increasingly, intraluminal filament MCAo in rats has been preferred over other species and models. Although the use of recently developed transgenic mice has provided a potent new tool for stroke research, the use of intraluminal filament in mice is associated with a significant variability in infarct volumes and increased incidence of complications, which may be related to differences between strains and ages, or even to minor technical differences.

In order to validate a model of ischemic stroke in new transgenic mice, the effect of MCAo on tissue necrosis, formation of glial scar and development of regenerative process needs to be thoroughly assessed to determine the pathophysiological mechanisms following permanent or temporary MCAo and thus the potential effect of therapeutic interventions.

Here we compare two different models of focal ischemic stroke generated by MCAo cauterization or ferric chloride induced thrombosis. BALB/Ca-RAG2^-/-^γc^-/-^, immune-deficient mice are used to develop a model permissive for cell therapy with human cells.

## Methods

### Animals and Surgical Procedures

Adult (7week-old) male BALB/Ca-RAG2^-/-^γc^-/- ^mice were housed in a temperature-controlled room with access to food and water *ad libitum *. All experiments were performed in accordance with "Guide for the Care and Use of Laboratory Animals", and animal procedures were approved by the University of Navarra.

**Figure 1:**
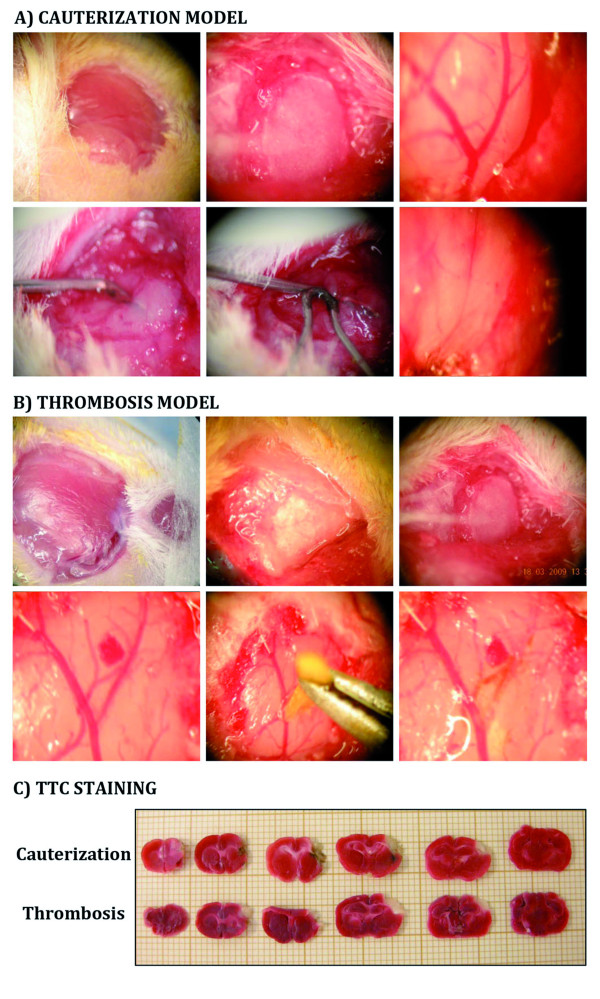
**Description of cauterization and thrombosis stroke models**. **A-B) **Photomicrographs of the surgical procedure in cauterization and thrombosis stroke models respectively, leading to the blood flow cessation. **C) **TTC staining.

Mice were anesthetized with 80-100 mg/kg ketamine and 10 mg/kg xylazine. A small craniotomy was performed, the dura was excised, and the middle cerebral artery (MCA) exposed. Cauterization model: Once the MCA was exposed a metallic forceps was introduced surrounding the artery, then the cauterizer touched the forceps for several minutes, producing the artery cauterization (Figure 1A) [6]. The control group underwent similar operative procedure; however the cauterizer instead of touching the forceps was placed close to the forceps. FeCl_3 _thrombosis model: Thrombus was induced by 1min topical application of a small strip of filter paper soaked with 20% FeCl_3 _with the adventitial surface of the vessel (Figure 1B) [7,8]. In control group 0.9% saline was used. FeCl_3 _produces endothelial damage and denudation, which leads to thrombin-fibrin and platelet dependent thrombus formation probably primarily via the collagen/GPVI axis [9,10].

### Behavioral test

Behavioural analyses were performed 24hr, 48hr and 96hr after stroke according to the modified neurological severity score test (mNSS) proposed by Chen [11]. Neurological function was graded as 0 to 14 (normal score, 0; maximal deficit score, 14) by which motor and sensory function; balance impairment and reflex abnormality were evaluated (Table 1).

**Table 1:** Modified Neurological Severity Score Test (mSCC) (modified from Chen et al, 2001).

**Raising rat by tail **(normal = 0; maximum = 3)	(3)
Flexion of forelimb	1
Flexion of hindlimb	1
Head moved > 10° to vertical axis within 30 s	1
**Placing rat on floor **(normal = 0; maximum = 3)	(3)
Normal walk	0
Inability to walk straight	1
Circling toward the paretic side	2
Falls down to paretic side	3
**Beam balance test **(normal = 0; maximum = 3)	(6)
Balances with steady figure (>60 s)	0
Grasps side of the beam	1
Hugs beam and 1 limb falls down from beam	2
Hugs beam and 2 limbs falls down from beam, or spins on beam (>30 s)	3
Attempts to balance on beam but falls off (>20 s)	5
Attempts to balance on beam but falls off (>10 s)	6
Falls off, no attempt to balance or hang on the beam (<10 s)	
**Reflex absence and abnormal movements **(normal = 0; maximum = 2)	(2)
Pinna reflex (head shaken when meatus is touched)	1
Corneal reflex (eye blink when cornea is lightly touched with cotton)	1
**Maximum Points**	**14**

### TTC staining

24hr after stroke, mice were sacrificed and their brains sliced into 6 serial coronal sections of 1 mm thickness. Slices were stained with 2% TTC at 37°C for 30min.

### Tissue processing, staining and morphometric analysis

Four animals for each time point were sacrificed at different time points (48hr, 1wk, 2wk and 4wk) after stroke. Animals were perfused intracardially with 4% paraformaldehyde (PFA). Brains were removed, postfixed overnight (ON) and cryoprotected in 15% sucrose ON. Subsequently, brains were frozen and coronally cryostat-sectioned at 14 μm. 10 serial series (containing 1 section out of every 10) were prepared for volume analysis and immunohistochemical quantifications (Additional file 1).

#### Immunostaining

Serial sections were incubated in blocking solution, followed by an ON incubation at 4°C with the primary antibody: rabbit anti-GFAP 1:250 (DakoCytomation); mouse anti-BrdU, 1:50 (DakoCytomation); rat anti-CD31, 1:500 (BD-Biosciences) or goat anti-Iba1, 1:200 (Abcam). For GFAP immunostaining sections were rinsed and EnVisionTM-HRP conjugated system (DakoCytomation) was used as secondary reagent. Afterwards sections were incubated 30min in 0.1% Sirius-red, dehydrated and mounted in DPX. For immunofluorescence, sections were washed and incubated for 1hr with the appropriated secondary antibody: mouse-Cy3 or rabbit-FITC or goat-Cy3; 1:200 (Jackson-Immunoresearch).

#### Cell proliferation

To evaluate the activation of SVZ proliferation, a single dose of BrdU (50 mg/kg) was injected 2hr before sacrifice (n = 4). For BrdU immunostaining sections were incubated with 2N HCl 30min at 37°C and rinsed in 0.1 M borate buffer pH 8.5 for 15min. After this pretreatment sections immunostaining was carried out following the standard procedure mentioned above. BrdU^+ ^cells in the SVZ were counted blindly in (5-7) 14 μm coronal section per animal, spaced 140 μm apart, under high magnification. Results were expressed as the average number of BrdU^+ ^cells per section.

#### Neovascularization

Blood vessel density estimation was performed in the ischemic boundary region using an Axioplan2-Zeiss automated microscope. This region was delineated from the edge of the pan-necrotic cystic cavity approximately 400 μm into the adjacent cortex. Within these boundaries four randomly fields at 10x magnification were analyzed. Blood vessel density was estimated as the percentage of CD31^+ ^area in 8 sections per animal, spaced 140 μm apart, in the ipsilateral hemisphere (n = 4).

#### Measurement of infarct volume

Infarct volumes were measured in 4 mice per stroke model 48hr after stroke. One of each 10 serial sections per animal was stained with 0.25% thionin. Similar levels were selected ranging from +2.0 to -3.0 mm from Bregma in the anterior-posterior axis, using the Paxinos stereotaxis mouse atlas. The area of infarction (necrotic tissue) and the area of both hemispheres were calculated by tracing the area on the computer screen. To reduce errors associated with processing of tissue for histological analysis, the area of infarction in each section was presented as the percentage of the infarct in comparison to the area of the contralateral hemisphere. The accuracy to delimit the area of infarction was further analyzed by EM.

### Tissue processing for semithin sections and electron microscopy

For electron microscopy (EM), animals were perfused with 2% PFA and 2.5% glutaraldehyde at different time points (48hr, 1wk, 2wk and 4wk) after stroke. Three animals for stroke model and for each time point were sacrified (Additional file 1). Brains were postfixed ON in the same fixative and cut into 200 μm coronal sections. Sections were post-fixed in 2% osmium tetroxide for 2hr, rinsed, dehydrated, and embedded in Durcupan. Semithin-sections (1.5 μm) were cut with a diamond knife and stained with 1% toluidine blue. Ultrathin-sections (60-70 nm) were cut, stained with lead citrate, and examined under a FeiTecnai Spirit electron microscopy. Semithin and ultrathin sections were obtained to analyze the ischemic and penumbra evolution as well as the activation of the SVZ at different time points after stroke.

For SVZ EM analysis, the SVZ was photographed in 10 consecutive pictures. Photo merges for each SVZ were performed with Photoshop. All the cells present in the SVZ were identified and their nuclei traced and painted using a different colour for each cell type in a separated layer with Photoshop tools.

### Statistical Analysis

Results are reported as the mean ± SD. Differences between means were determined by Student's t test, with P < 0.05 considered significant. The reproducibility of the stroke in thrombosis and cauterization stroke model were compared by the Sign-Test, with P < 0.05 considered significant.

## Results

The mNSS test was used to evaluate neurological deficits 24hr, 48hr and 96hr after MCAo in both models. However, no significant differences were observed between control and stroked animals. Thus, it was not possible to implement a functional test for any of the two models.

### Assessment of stroke

The surgical procedures for cauterization and thrombosis stroke models are depicted in figure 1A-B. Both models have the advantage that MCAo is confirmed directly through operative microscope and that the mortality rate was low (13.9% and 7.8% respectively). In all animals tested, physiological variables (temperature, pH, pCO_2_) remained within normal range through the observation period. To confirm the induction of stroke, TTC staining was performed 24hr after the procedure. In both models the infarction area is restricted to the cortex and no changes were observed at the contralateral hemisphere (Figure 1C). TTC staining pattern in stroked BALB/c wt mice was equivalent to that observed in RAG 2^-/- ^γC ^-/- ^mice (data not shown).

### Histopathological comparison between cauterization and thrombosis model

To compare the glial scar formation and the evolution of damaged tissue in both stroke models and to determine the best model for further analysis, brain semithin and ultrathin sections were analyzed at different time points after stroke (48hr, 1wk, 2wk and 4wk). The analysis would allow evaluating how different therapies would affect wound healing and regeneration after stroke in future studies.

**Figure 2:**
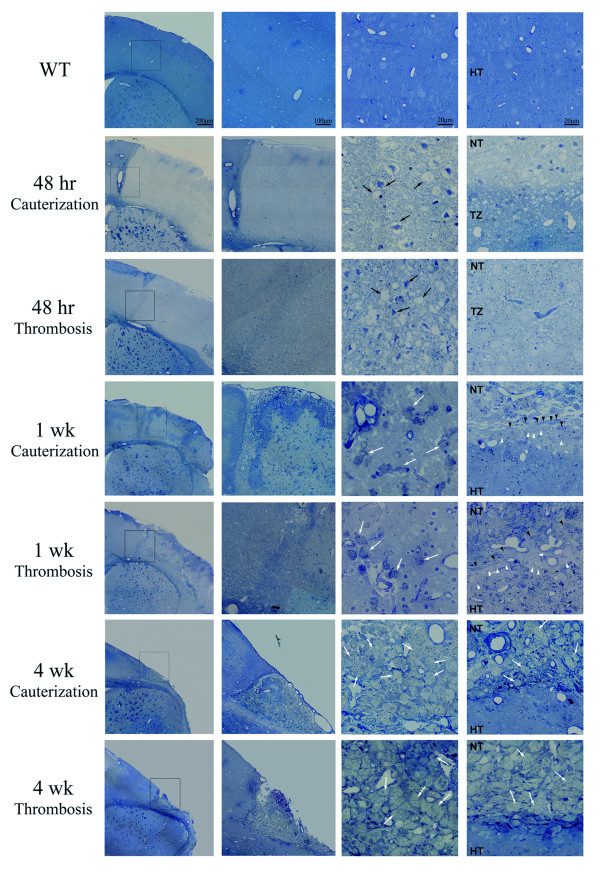
**Damage evolution and glial scar formation in cauterization and thrombosis models**. Photomicrographs of toluidine blue-stained semithin-sections at different time points after stroke. For each time point the first picture represents a panoramic view of the whole hemisphere. The area squared in this picture is depicted next at higher magnification. The last two represent details of the necrotic area and the boundary ischemic zone. Black arrows point piknotic cells and cellular ghosts. White arrows point to macrophages. Black arrowheads point to elongated cells and white arrows point to voluminous non-macrophage cells (TZ: transition zone; HT: healthy tissue; NT: necrotic tissue).

**Figure 3:**
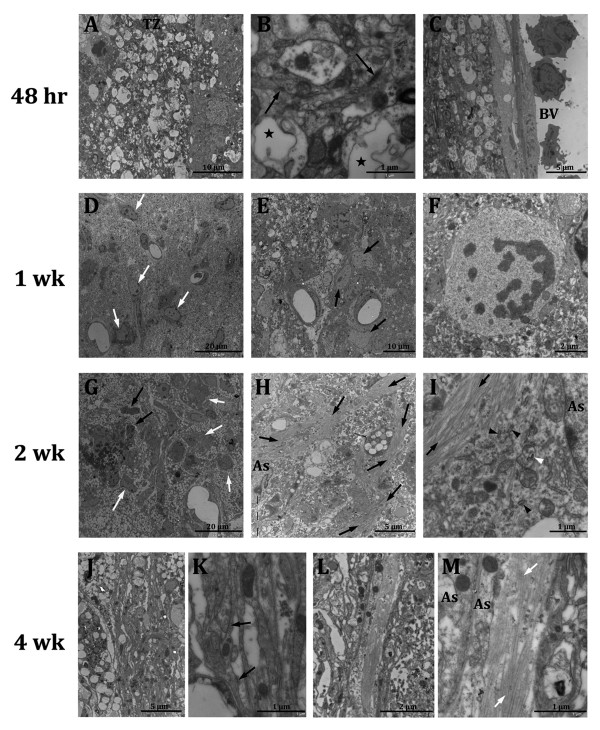
**EM details of damage evolution and glial scar formation**. Micrographs at different time points after stroke. At 48hr the transition zone (TZ) **(A) **is observed. Black arrows point to synaptic contacts and black stars indicate cellular ghosts **(B)**. **(C) **shows a detail of infiltrating neutrophils. At 1wk macrophages (white arrows) appear in the necrotic neuropil **(D) **and astrocytes (black arrows) delineate the lesion border **(E)**. In some cases mitoses of astrocytes are observed **(F)**. At 2wk after stroke, large macrophages (black arrows) and infiltrated neurotrophils (white arrows) occupy the majority of the lesion volume **(G)**. Hypertrophic astrocytes with plenty of intermediate filaments (black arrows) display lining the ischemic boundary **(H-I)**. Black arrowheads indicate rough endoplasmic reticulum cisternae and white arrowheads point to small dictiosomes. At 4wk after stroke, an imbricate layer of hypertrophied astrocytes composes the boundary of the ischemic zone **(J-K)**. At higher magnification these astrocytes appeared attached by tight junction (black arrows). **L-M **show a detail of astrocytes associated to collagen fibers, forming a base membrane (BV:blood vessel; As:astrocyte).

Morphological and pathological changes were similar in both models and included the development of a pale staining area of pan-necrosis after 48hr (Figure 2). The wound was composed of necrotic edematous tissue with shrunken neurons and neurons with a loss of cellular integrity. The limit between healthy and necrotic tissue was initially not well demarcated with a progressive loss of cellular integrity from the healthy tissue towards necrotic tissue (*penumbra *). These observations were confirmed by EM analysis showing necrotic areas characterized by the presence of pyknotic neurons and cellular ghosts, where synaptic contacts were no longer observed while there was abundant activated microglia (Figure 3A). However, in the transition zone between the necrotic area and the healthy tissue, more synaptic contacts and higher plasmatic membrane integrity were observed (Figure 3B). Due to inflammation numerous neutrophils were observed within the vascular lumina and associated with the blood vessel endothelium, close to necrotic brain tissue (Figure 3C). However, macrophage activation was not observed at this time point.

One week after stroke, infarcts were well delineated from the surrounding healthy area. Scattered foamy macrophages were present in the infarct area and voluminous non-macrophage cells with light cytoplasm close to elongated cells displayed parallel to the lesion (Figure 2). At EM level macrophage infiltration was confirmed, showing that necrotic neurophil was composed of cellular debris (Figure 3D). Voluminous cells were identified as hypertrophic astrocytes rich in intermediate filaments, which displayed parallel to the lesion delimitating healthy tissue from necrotic tissue. Sometimes, astrocytes were observed under mitosis (Figure 3E-F). An infiltrate of macrophages with abundant vacuolated cytoplasm occupied the majority of the lesion volume after 2 weeks (Figure 3G). An increase in hypertrophic astrocytes with large and thick cytoplasmic expansions were observed displaying parallel to the lesion border (Figure 3H-I). Some of these cells were also observed in inner locations close to vacuolated macrophages.

By 4 weeks, a large cavity had formed in the region occupied by necrotic tissue (Figure 2), although a small portion of necrosis and connective tissue persisted along the lateral aspects of the superficial portion of the lesion. Necrotic tissue is progressively removed while collagen fiber formation takes place; this led to a progressive increase in the size of the cavity. An imbricate layer of hypertrophied astrocytes attached to one another by tight junctions lined the cavity. In association with connective tissue elements, such as fibroblasts and collagen fibers, astrocytes processes form a base membrane, with abundant tight junctions and hemidesmosomes, which resemble the normal membrane superficial glia membrane (Figure 3J-M). Activated macrophages and inflammatory response have decreased due to necrotic tissue removal.

Examination of ipsilateral hemispheres from sham-operated animals and contralateral hemispheres of MCAo animals provided appropriate controls for histological changes observed following MCAo. Inflammation, glial scar formation and damage evolution underwent equivalent progress in BALB/c wt mice compared to RAG 2^-/- ^γC ^-/- ^mice in both stroke models (data not shown).

**Figure 4:**
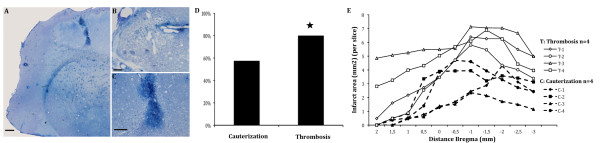
**Thrombosis vs cauterization stroke model . A-C) **Photomicrographs of toluidine blue-stained semithin sections showing unspecific damage in cauterization stroke model (A scale-bar = 200 μm, B scale-bar = 50 μm and C scale-bar = 100 μm). **D) **Quantification of stroke incidence (*P < 0,05). **E) **Distribution of the ischemic lesion induced by cauterization (C) stroke model (dotted lined, n = 4: C1-4) and thrombosis (T) stroke model (continuous line, n = 4: T1-4).

Although both models recapitulate similar mechanisms and pathways in the damage tissue evolution, there were some differences between them: first, cauterization induced a non-specific injury in the cortex, leading to macrophage infiltration and damaged tissue degradation (Figure 4A). In addition, numerous studies have shown an activation of endogenous brain stem cells in the SVZ after neuronal injury [12]. Unspecific injury induced by cauterization could activate cell proliferation in the SVZ, thereby masking the effects produced by stroke, which also activate SVZ proliferation and the inflammatory processes [13-17]. Second, while cauterization produced a stroke in 57.5% of the animals, thrombosis model produced 80%, which supports thrombosis as a more reliable model (Figure 4B) (n = 40 mice per stroke model). Finally, the infarct volume distribution was more homogeneous in thrombosis model compared to cauterization model (Figure 4C). For these reasons, thrombosis model was chosen for further analysis.

### Thrombosis stroke model characterization

Next and in order to characterize the healing process after stroke immunostaining against collagen (Sirius-Red), astrocytes (GFAP), vascular endothelial cells (CD31) and microglia (Iba1) was performed.

**Figure 5:**
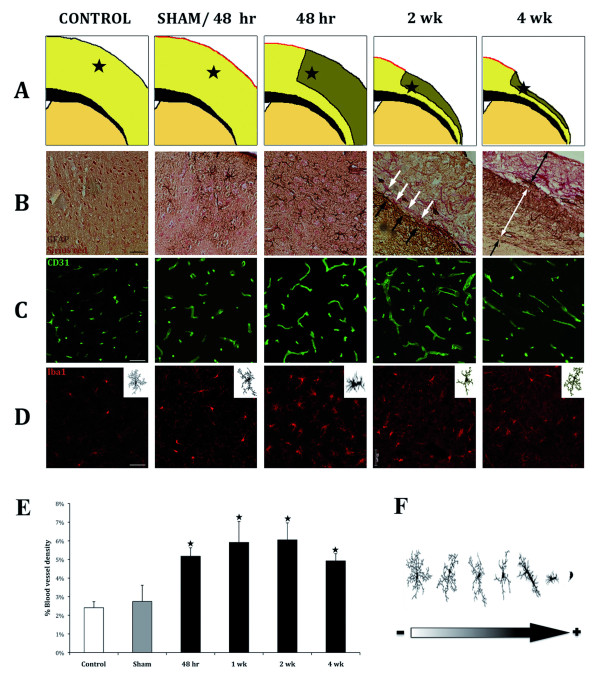
**Immunohistochemical analysis of tissue damage evolution after stroke**. **A) **Diagram of the progressive tissue loss after stroke. **B) **Immunostaining against GAFP (brown) and counterstained with Sirius-red (reddish) to show the glial scar formation with time; **C) **Immunostaining against CD31 (green), which point an increase in blood vessel; **D) **Immunostaining against Iba1 (red) to study microglia activation (scale-bar = 50 μm); **E) **Quantitative analysis of neovascularisation after stroke. **F) **Scheme of different stages of microglia activation from less activated towards more activated.

An increase not only in astrocytes and GFAP filaments, but also in collagen fibers was observed with time. While sham group presented very low macrophage activation due to meningeal breakage, a clear activation with numerous astrocytes is observed 48hr after thrombosis induction. At 2wk, GFAP labeling is observed lining the lesion border while collagen fibers are secreted parallel to the lesion. At 4wk three tissue layers were observed; the outer one is mainly composed by collagen fibers, the intermediate contains many hypertrophic astrocytes and the inner one represents uninjured tissue (Figure 5B).

Immunostaining against CD31 was performed to analyze blood vessel formation after stroke. Qualitative and quantitative analysis show an increase in blood vessel density at all time points analyzed compared to control or sham mice. Interestingly, blood vessels grew and branched towards the scar tissue (Figure 5C, E).

Under normal conditions, microglia are ramified, showing a large number of processes. Activated microglia take a rounded amoeboid shape, reducing the length and number of processes (Figure 5F). Iba1 immunostaining showed different levels of activation of microglia after stroke. Sham mice showed an increase in microglia somata size in comparison with control animals, likely related to the meningeal breakage. 48hr after stroke showed maximum activation of microglia as indicated by the rounded and amoeboid morphology, which was coincidental with maximal degeneration and degradation processes. At later time points microglia become progressively less activated (Figure 5D). This could be due to cavitation, as necrotic tissue is progressively phagocyted and almost completely removed by 4 weeks.

### Activation of SVZ proliferation after stroke

**Figure 6:**
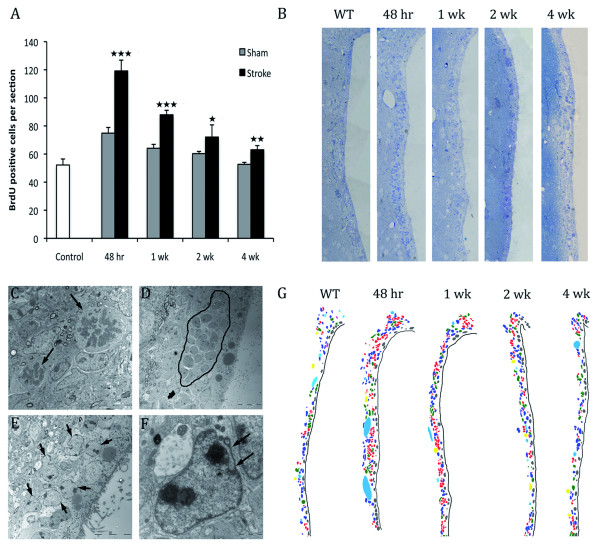
**SVZ activation after thrombotic stroke. A) **SVZ quantification of BrdU^+ ^cells at different time points (48hr, 1 wk, 2wk and 4wk). Cells were counted in the ipsilateral side of ischemic brains and in non-ischemic brains. Data are represented as the mean ± SD (n = 5)(Student's t test ***P < 0,0005; **P < 0,005 and *P < 0,05). **B) **SVZ semithin sections indicating activation of the proliferation. **C-F) **Transmission electron micrographs of common events in activation of proliferation. **C **shows two mitoses pointed by arrows. **D **shows a robust migrating chain encircled by a line. **E **shows an accumulation of augmented astrocytes, pointed by arrows. **F **highlights an invaginated astrocyte. **G) **Coloured reconstruction based on EM photomerged images showing the different cell types increase after stroke. Dark-blue: astrocytes, green: Type-C cells, red: neuroblasts, grey: ependymal cells, yellow: neurons, violet: microglia and blue: blood vessels. There is a remarkable activation 48hr after stroke with an increase in the number of type-B, type-C and type-A cells.

Previous studies have demonstrated that stroke induced by MCAo leads to an increased SVZ cell proliferation, from rodents (10-14, 27) to human [18]. To determine if this feature was present in the thrombotic model, BrdU was injected on the day of sacrifice. BrdU quantifications showed a significant increase in cell proliferation at all time points analyzed in animals suffering from stroke compared to sham mice. This increase peaks at 48hr after stroke and decreases with time (Figure 6A).

Cell proliferation was confirmed by the analysis of the upper third of the SVZ where a notable cell increase was observed after 48 hours. This increase appeared as a layer of 4-7 cells, which extends through the ventricular wall. Such activation is observed up to 1wk after stroke (Figure 6B) with a progressive decrease. Four weeks after stroke the SVZ looked similar to control mice (Figure 6B). Ultrastructural reconstruction of the upper third of the SVZ was performed to determine the contribution of the different cells types to the SVZ activation. At 48hr and 1wk after stroke a notable increase in the number of type-B cells (astrocytes), type-C (transient amplifying cells) and type-A (migrating cells) was observed with a subsequent decrease throughout time (Figure 6G). However, no differences were observed in ependymal cells at any time point analyzed.

Frequent mitosis (Figure 6C) as well as numerous type-C and type-A cells arranged in enlarged chains (Figure 6D) at 48hr and 1wk after stroke indicates that SVZ proliferation is highly activated. All the mitoses observed belonged to type-B, C and A cells. Ependymal cells were never observed undergoing mitosis. In addition, numerous enlarged astrocytes were observed close to each other with many cytoplasmic organelles, mainly polyribosomes (Figure 6E). Interestingly, these astrocytes presented less intermediate filaments, their nuclei showed 2-3 prominent nucleoli and frequently the invaginated nuclear membrane flattened and adopted a rod-like appearance (Figure 6F).

## Discussion

Several models of global and focal stroke have been developed in many animal species. Overall, focal models with preference for MCAo, are regarded as the best approach, given that focal ischemia affecting the MCA territory is the most frequent type of stroke in humans. Nevertheless, the use of transgenic mice to investigate pathophysiological mechanisms after stroke requires the development of a reproducible and relevant model for stroke in mice mimicking the large cerebral thromboembolic stroke in human.

In the present study, we have compared two models of focal cerebral ischemia in mice: FeCl_3 _thrombosis stroke model and cauterization model. Despite similar infarct sizes and mortality, thrombosis model showed advantages over cauterization model related to the reliability of the model. Furthermore, cauterization produced a permanent ischemia, while the thrombosis model could be amenable to the use of thrombolytic agents leading to reperfusion.

Although both stroke models fail to reproduce the type of stroke that occur in people, these models mimic the type of injury, the post-lesion effects as well as the pathophysiological processes after stroke (inflammation, oxidative stress, scarring....), which are key of importance for the final stroke outcome. Thereby these stroke models are useful to evaluate damaged tissue evolution after stroke and the effect of stroke therapies in this area. We intend to provide a morphological method for stroke evaluation.

Mice exhibit notable interstrain differences in infarct after MCAo [19-22]. Strain variability in both cerebral vasculature and neuronal vulnerability can contribute to these differences. Moreover, mouse strains have differences in many gene system expression [23]. Thus, the use of new transgenic mice requires a detailed characterization of the response to the insult. BALB/Ca-RAG2^-/-^γc^-/- ^mice were selected in this study to validate a reproducible mouse model of stroke, which would allow the use of human cells in an immune permissive milieu allowing for cell therapy studies. In comparison with other immunedeficient mice, BALB/Ca-RAG2^-/-^γc^-/- ^mice have no T and B cells, no NK activity and impaired DC function [24,25], which make them suitable for further cell transplant experiments, with cells from different origins and species, including human cells. A limitation in the model could be the analysis of inflammation at very early stages after stroke, given the transgenic characteristics of these mice, although no differences were observed at the time points analyzed (1 week-4 week) after stroke, compared to BALB/c wt mice. However, these mice also provide the possibility to reconstitute the immune system with human cells (humanized mice) [26], which would be useful to test the role of the human immune system in stroke.

Following the acute stroke phase, the tissue undergoes a remodeling process that results in (astro)glial scar formation and cavitation. The transition zone that develops after 48 hours surrounding the necrosis area shows a progressive loss of cellular integrity from healthy tissue towards necrotic tissue. This region may be non-functional but viable and potentially reversible hours after insult. Secondary energy failure and injury evolve in this zone up to one week, where the limits between necrotic and healthy tissue appear better delineated. In the ischemic border zone surrounding the evolving infarct, inflammatory responses and apoptotic programs are activated that further contribute to injury development [27-29]. In fact, 2 and 4 weeks after stroke astrocytes were observed in inner positions close to the fibrotic scar, suggesting the possibility that cavitation process continues taking place after these time points. Our results provide an exhaustive analysis of the histopathological changes that occur after stroke in the ischemic boundary zone, which are of key importance for the final stroke outcome. Therefore, therapies should be conducted to minimize cavitation process firstly by protecting the transition zone avoiding the increase in necrotic tissue and secondly, protecting the surrounding healthy tissue from the continuous scarring process.

It has been described that age plays an important role in the recovery of the brain from insult, since microglia and astrocytes increase both in number and reactivity in normal older subjects. Badan and colleagues and DiNapoli and colleagues described that the glial scar develops abnormally early in the infarcted region of aged rats when compared to young rats, thereby hampering the functional recovery of surrounding nervous tissue into a greater extent [30,31]. Aged animals recover more slowly and less completely than young animals. Although remodeling and neuronal degeneration are accelerated in aged rats, there is no consensus in the ultimate extent of brain cell loss whether it is or not significantly different from aged to young animals [32-36]. It could be relevant describe and compare deeply the remodeling and scar formation process in young and aged animals and test stroke therapies in stroke models in aged animals, given than age could be the clue for some differences between experimental and clinical studies in aged humans [37].

Stroke in mice induces an increase in proliferation in the SVZ as well as migration of newly generated neuroblasts toward the damaged are as an attempt of self-repair [13,14,16,17,38]. Thrombotic stroke in our mice model also produced a stimulation of endogenous neural precursor, which was observed by the presence of numerous mitoses and the increase in type-B and type-C cells and consequently in type-A cells and BrdU quantification. Interestingly, type-B cells presented some morphologic changes, such as a decrease in intermediate filaments and an increase in ribosomes. These changes point to a transition from type-B cells to type-C cells. Despite SVZ activation and migration toward the damaged area, the vast majority of neuroblast die primarily by apoptoptic mechanism, and only a small proportion are able to survive and differentiate into neurons [13]. Neuroblast death may be related to the fibrotic scar boundary environment that cannot provide the appropriate trophic support for neuroblast survival as well as their integration within the tissue. Therapies aimed to improve environmental conditions in the scar boundary should be developed first to protect tissue from degeneration and last to potentiate neuroblasts survival and integration.

Unfortunately, although both stroke models present tissue necrosis and cavitation process with time, no neurological defects were observed with the modified neurological severity score test (mNSS). We think that no behavioral deficits were found after ischemic insult because of the type of lesion induced. This lesion is small and localized to the parietal cortex. By one hand mice recover very quickly after surgery and by other hand the other intact hemisphere could be supplying the neurological functions perhaps through callosum connections and plasticity, as the lesion area is small and the corpus callosum is not damaged. Another possible explanation is that although mNSS is one of the most used neurological tests to evaluate the neurological outcome after stroke, in this case as the lesion is small and area-restricted, this test could not be sensible enough due in part to the collaboration of the other hemisphere, requiring another one more demanding or precise.

## Conclusions

In summary, we compare thrombosis and cauterization stroke model, giving evidence in favor of the thrombosis model. The major advantages of this model are 1) its potential use in mice models, including transgenic animals; 2) the possibility to study the effects of cell based therapies with cells from different origins and species, even human, avoiding the rejection problem or even in combination with neuroprotective compounds or thrombolytic agents. The histological and ultrastructural analysis performed should also be very useful to determine the potential effects of different cell therapies in the remodeling process including fibrotic glial scar formation and cavitation as well as the role of these therapies in neurogenesis after stroke.

## Authors' contributions

JMGV and FP conceived the project, obtained financial support and gave final approval to the manuscript; GA performed the cauterization stroke model and took care of the animals. SML, TL performed the thrombosis stroke model, perfused the animals and the immunohistochemical analysis. CJ prepared the macro design for the blood vessel density estimation. MRR and MCN performed the electron microscopy studies. MGP, UGP and MSSP planned the experiments and prepared the manuscript. All authors read and approved the final manuscript.

## Competing interests

The authors declare that they have no competing interests.

## Supplementary Material

Additional file 1 **Experimental design**. Diagram with the groups, animal per group and time points analyzed. Click here for file
