# Supplementary figures and images for: Histological and ultrastructural comparison of cauterization and thrombosis stroke models in immune-deficient mice
Source: J Inflamm (Lond). 2011 Oct 18;8:28. doi: 10.1186/1476-9255-8-28 (PMC3221623; doi:10.1186/1476-9255-8-28)

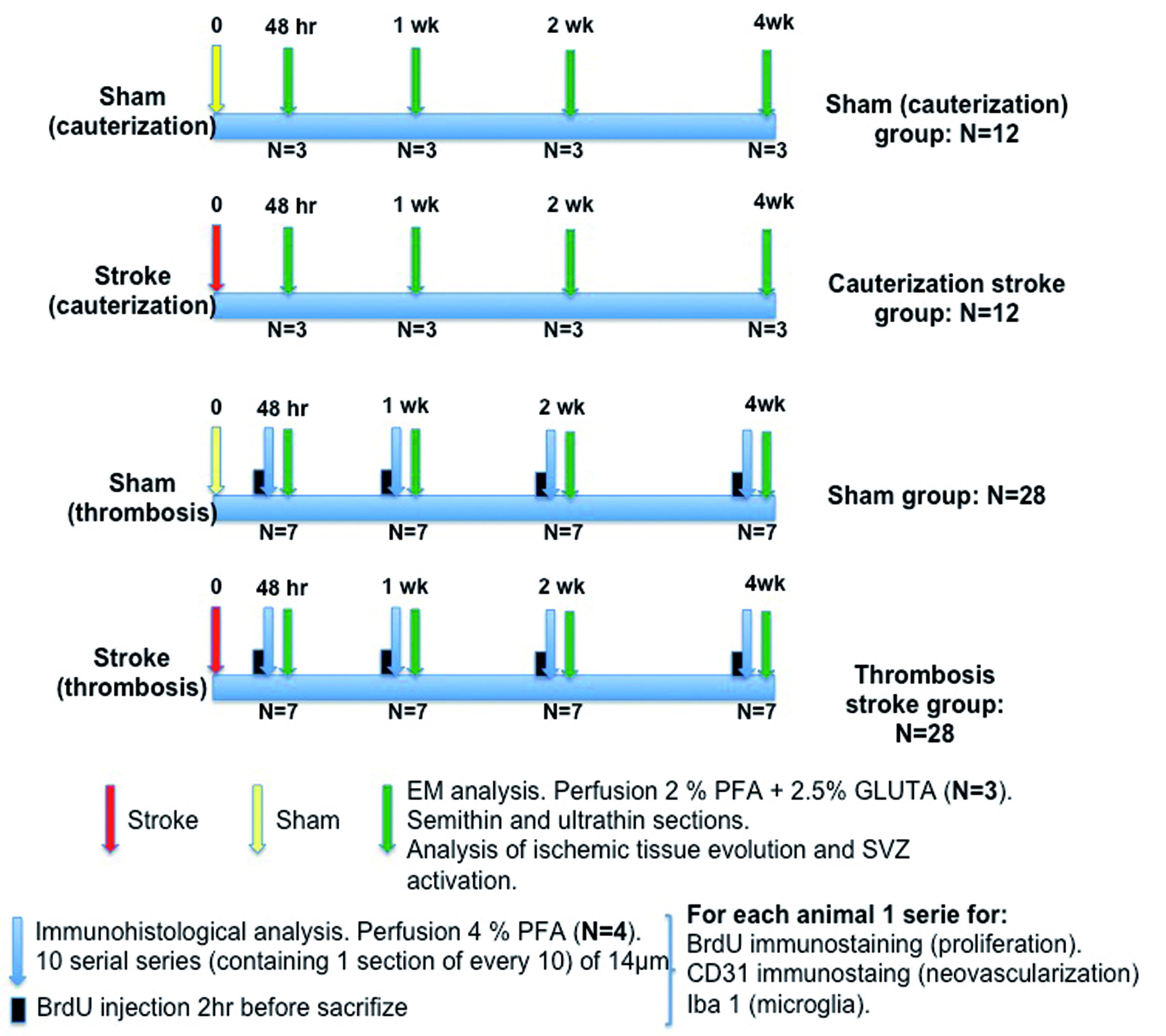

Supplement: Additional file 1 — Experimental design. Diagram with the groups, animal per group and time points analyzed. [file 1476-9255-8-28-S1.JPEG]
